# Contrasting Patterns of Species Richness and Functional Diversity in Bird Communities of East African Cloud Forest Fragments

**DOI:** 10.1371/journal.pone.0163338

**Published:** 2016-11-17

**Authors:** Werner Ulrich, Luc Lens, Joseph A. Tobias, Jan C. Habel

**Affiliations:** 1 Nicolaus Copernicus University in Toruń, Chair of Ecology and Biogeography, Pl-87-100 Toruń, Poland; 2 Ghent University, Department of Biology, Terrestrial Ecology Unit, B-9000 Ghent, Belgium; 3 Department of Life Sciences, Imperial College London, Silwood Park, Buckhurst Road, Ascot, Berkshire, SL5 7PY, United Kingdom; 4 Technische Universität München, Department of Ecology and Ecosystem Management, Terrestrial Ecology Research Group, D-85354 Freising, Germany; Centre for Cellular and Molecular Biology, INDIA

## Abstract

Rapid fragmentation and degradation of large undisturbed habitats constitute major threats to biodiversity. Several studies have shown that populations in small and highly isolated habitat patches are prone to strong environmental and demographic stochasticity and increased risk of extinction. Based on community assembly theory, we predict recent rapid forest fragmentation to cause a decline in species and functional guild richness of forest birds combined with a high species turnover among habitat patches, and well defined dominance structures, if competition is the major driver of community assembly. To test these predictions, we analysed species co-occurrence, nestedness, and competitive strength to infer effects of interspecific competition, habitat structure, and species′ traits on the assembly of bird species communities from 12 cloud forest fragments in southern Kenya. Our results do not point to a single ecological driver of variation in species composition. Interspecific competition does not appear to be a major driver of species segregation in small forest patches, while its relative importance appears to be higher in larger ones, which may be indicative for a generic shift from competition-dominated to colonisation-driven community structure with decreasing fragment size. Functional trait diversity was independent of fragment size after controlling for species richness. As fragmentation effects vary among feeding guilds and habitat generalists, in particular, tend to decline in low quality forest patches, we plead for taking species ecology fully into account when predicting tropical community responses to habitat change.

## Introduction

Habitat fragmentation has profound and mainly negative effects on the long-term viability of indigenous animal and plant species [1–2], in particular in historically stable ecosystems such as tropical rainforests [3–5]. While habitat fragmentation mainly causes a decline in species richness at the regional level [6–7] (but see counterexamples in Schmiegelow et al. 1997 and Debinski & Holt 2000 [8–9]), it may trigger increased species richness (species density sensu Gotelli & Ellison 2002 [10]) and abundances at the local (i.e. fragment) level [11–13]. Such a “crowding” effect (sensu Collinge & Forman 1998 [14]) might intensify species interactions, particularly interspecific competition for resources and space [15–16], while at the same time reducing population sizes and genetic diversity at the species level [17]. Ultimately, this may affect long-term survival, food web structures, and ecosystem functioning [18].

Variation in species density, the average number of species per unit area, is closely related to the concept of species-area relationships (SAR). Given the common power function model of the SAR S = S_0_A^z^ with S_0_ being the average number of species per unit area and z being the scaling constant [19] the species density S_A_ becomes $S_{A}=\frac{S}{A^{z-1}}=constant$ and is expected to be independent of patch area. The crowding effect [14] predicts increased species density in smaller habitat fragments. However, previous simulation studies showed that species densities in homogeneous landscapes only moderately increase at small patch sizes [20]. Hence, a positive deviation of observed species densities from those predicted under the neutral model may indicate a “crowding effect”, while a negative deviation may indicate that species numbers are mainly limited by environmental factors that correlate with fragment size.

Classic competition-based community assembly models [21] predict fragmented landscapes to exhibit a scattered pattern of species occurrences in which species with similar ecological niches occur in a segregated manner [22–24]. Indeed, competition-mediated species segregation has been found in a number of studies on landscape fragmentation [16, 25, 26]. However, species segregation is not the only possible outcome of fragmentation. If resource availability and mutualistic interactions outweigh competitive effects, habitat filtering may also lead to an aggregated pattern of species occurrences [26, 27]. Further, if key resources are unevenly distributed among fragments, patterns of species occurrences may follow the respective gradient in resource availability leading to a nested pattern of species occurrences [23, 28] where species assemblages in resource poor fragments are true subsamples of those in richer ones [28]. Due to the fact that nestedness and species segregation are opposing patterns, community organisation will often be intermediate between both extremes, depending on the respective pay-offs between species competition and habitat filtering [24]. Finally, ecological demands and behaviour of species may strongly affect species′ sensitivity to rapid changes in the habitat configuration. While habitat specialists (species with specific habitat demands and restricted movement behaviour) are assumed to suffer strongly under ongoing habitat degradation, habitat generalists (which can be found in various habitat types) are assumed to be able to better adapt to environmental changes [29].

Mechanisms underlying community assembly are still discussed even after more than half a century of research in the field. Standard analyses of community assembly are based on species occurrence and absence, however, these data are only rarely linked to trait- and environmental variation. When aiming to uncover mechanisms and constraints behind the pattern of species co-existence, there is a clear need to link the geometry of species occurrences with environmental and species functional trait data which can be expected to replace classic co-occurrence analysis that have been dominated the field since the pioneering work of Diamond (1975) [30]. Along these lines, Ulrich et al. (2014) [31] and Soliveres et al. (2015) [32] recently introduced a novel Markov chain-based approach to examine the frequency of intransitive competition in real-world communities and how they affect community diversity. We here apply this method to assess the role of intransitive competition on species coexistence in forest bird communities within and among 12 tropical forest fragments that vary in patch size and habitat quality. The indigenous forest of the Taita Hills of south-east Kenya, the northernmost outlier of the Eastern Arcs and part of the Eastern Afromontane biodiversity hotspot, has been subject to rapid loss, degradation and fragmentation of pristine habitats over the past decades (further details see material and methods) [33]. Despite this transition, indigenous forest remnants still harbour a typical cloud forest avifauna, including many endemic and endangered forest habitat specialists, but also a large number of habitat generalists that also occur in the non-indigenous landscape matrix.

The Taita bird community has been studied intensively over the past two decades, resulting in well supported knowledge about species richness, abundance, and ecological demands. Furthermore, the land use history of the Taita Hills is very well documented, and this combined information offers a strong framework to study how tropical avian communities are shaped in relation to species and landscape traits. Making use of this information, we here test the following three hypothesis: (i) Avian species density increases with decreasing fragment size, resulting in a high proportion of species surviving in forest fragments relative to intact forests; (ii) Species co-occurrence among small forest remnants is aggregated due to crowding effects; and (iii) Habitat specialists respond more strongly to habitat degradation compared to habitat generalists.

## Material and Methods

### Taita Hills study region

The Taita Hills cover an area of around 250 km^2^ and are geographically isolated from other mountain blocks to the south (90 km to the Usambara Mts.) and the north (80 km to the Chyulu Hills) (S1 Appendix). Semiarid plains in either direction constitute a strong dispersal barrier for species that depend on moist and cool cloud forest habitat, and this resulted in high levels of endemicity [34–35]. Degradation and fragmentation of the Taita forests started long before the colonial era, when slopes were cleared for agriculture up to the head of the streams [36]. Large-scale forest loss occurred during railway constructions between 1898 and 1924, while in more recent times, forest cover markedly decreased between 1955 and 2004. Even though half of the original indigenous forest has currently been lost, airborne remote sensing of spatio-temporal changes in forest cover [33] revealed that the total forest cover in the Taita landscape remained about the same between 1955 and 2004, mainly due to planting of exotic trees on rocky, barren or eroded areas, secondary bushlands and abandoned agricultural land. In addition to indigenous forest loss, the remaining patches also decreased in forest quality due to pit-sawing, charcoal manufacturing, firewood collection, pole removal and grazing [33]. Three larger forest fragments (Chawia (90 ha), Ngangao (147 ha) and Mbololo (179 ha)), nine smaller ones (< 15 ha), and several tiny patches of indigenous forest remained embedded in a fine-grained mosaic of human settlements and small-holder cultivation plots [33]. Small forest fragments, in particular, continue to suffer from cattle grazing and other forms of habitat disturbance, while the three larger forest fragments vary in the degree of habitat degradation too, being highest in Chawia forest, intermediate in Ngangao forest, and lowest in Mbololo forest [37].

Land-cover information was derived from airborne true-colour images, converted to orthomosaics at a spatial resolution of 0.5 m [33]. Brightness variations were removed by corrections for light falloff and bidirectional effects using the methods developed by Pellikka (1998) [38] after which frames were mosaicked using the EnsoMOSAIC [39]. The resulting mosaics were orthorectified, projected to Transverse Mercator projection with a Clarke 1880 [40] spheroid and Arc 1960 datum, and resampled to 0.5 m ground resolution. The resulting geometric accuracy was within 2 m as verified in the field using GPS. The land cover model was subsequently ground-truthed, revised and fine-tuned during field visits in 2007 and 2008, confirming the correct remote-sensing classification of large patches of closed—canopy forest, exotic plantations, and non-forested habitat [33]. Based on this land cover model, we calculated the following four landscape characteristics using Fragstats v. 3.3 and ArcView 3.2 (ESRI 2013): (i) indigenous forest patch size, (ii) indigenous forest patch perimeter; (iii) percentage of closed-canopy forest cover within 800 m of each indigenous forest patch, and (iv) patch proximity, a distance-weighted, area-based isolation index (PPI) [40]. We related observed species richness to these landscape characteristics to test the first and third hypothesis.

### Bird assessments

Understorey bird community metrics were derived from a long-term (1996 to 2010) bird ringing program using standard mist-netting procedures as described in Karr (1981) [41]. Mist netting was conducted in collaboration with the Ornithology Section of the National Museums of Kenya, Ornithology Section. Permission for bird collection was issued by the National Museums of Kenya. Permits to access the forest fragments were provided by the Kenyan Forest Service. As endangered, Taita endemic bird species were involved in this study, its collection was approved and mainly conducted by members of the ethics committee of the National Museums of Kenya personally (P Njoroge, RK Mulwa, O Kioko). Birds were collected using mist-nets, which were regularly controlled, to prevent any negative effects on trapped birds. This activity was approved by the animal ethics committee of the National Museums of Kenya. Mist-net lines were operated in one to seven plots per fragment (depending on fragment size) and were evenly spaced out to sample entire plots, while net positions, net lengths (120m/plot) and daily trapping efforts (06-18h) were kept constant between trapping sessions. Nets were routinely checked at 30-minute intervals so as to promptly remove, process, and release the birds. Time intervals between subsequent ringing sessions varied between 1.0 and 4.6 months, and the number of ringing sessions per fragment ranged between 20 and 32 over the 15 year study period. While mist nets are regarded as likely the best technique for assessing the relative abundances of tropical understorey birds [41, 42], habitat modifications such as removal of canopy trees and clearing of the understorey, in particular, may alter flight height of some species, thereby changing their susceptibility to mist-net capture [43]. To minimize this possible bias in the assessment of species richness, we restricted our analysis to the understorey bird community, i.e. species that are reliably caught in mist nets. Therefore, our data, covering 15 years of bird observation, are believed to be highly appropriate to assess total species richness and as well as relative abundances in the smaller fragments sampled with identical sampling effort.

For each bird species we assembled data on body mass (g), average bill culmen length, depth and width (cm), tarsus, tail, and wing length (cm), and average hand-wing index. The dominant principal component of the three bill measures was used to assess bill characteristics, and the respective dominant eigenvector of the wing, tarsus, and tail measures served as a proxy to the type of locomotion [44]. Following Claramunt et al. (2012) [45], we used the hand-wing index to quantify dispersal ability. Each species was assigned to one of four feeding guilds, insectivore, seed-eater, fruit-nectar feeder, and omnivore. We also assembled dietary (coded into ten categories) and foraging stratum preferences (coded into eight categories), respectively [46]. To reduce dimensionality, we calculated the dominant principal components of the matrices and used these in subsequent analyses. The complete species list of bird species, respective abundances per forest fragment, and species traits are given in the S1 Appendix.

### Statistical analysis

Analyses were based on matrices containing species relative abundances with species in rows and forest fragments in columns. We composed matrices for each fragment separately and then grouped the three larger (> 90 ha) and nine smaller (<15 ha) fragments in two additional matrices. We calculated the following three metrics of species co-occurrence. First, we estimated species segregation among fragments (negative species associations) by the C-score [24, 47, 48] that is a normalized count of the number of checkerboard submatrices ({{1,0},{0,1}} or {{0,1},{1,0}}). As an auxiliary metric of species segregation that focuses on the pattern of species turnover among sites we used the standard proportional turnover ${\text{beta}}_{\text{P}}=1-\frac{\text{alpha}}{\text{gamma}}{\text{beta}}_{\text{P}}=1-\frac{\text{alpha}}{\text{gamma}}$; where alpha refers to the average richness per fragment site and gamma tom the total observed species richness [49]. Third, we performed a nestedness analysis to identify gradients in species occurrences and richness across fragments [23] using the NODF (nestedness by overlap and decreasing fill) metric of Almeida-Neto et al. (2008) [48]. For the nestedness analysis, rows were always sorted according to species occurrence totals. Finally, we calculated the functional diversity of each feeding guild based on five measured functional traits (bill characteristics, body size, dispersal, locomotion, stratum) using the functional attribute metric FAD [49, 50], a measure of total trait space encompassed by the species of a given community calculated as the sum of the Euclidean distances between species in trait space. For comparability, trait expressions were Z-transformed prior to calculation. Co-occurrence analyses were done using the freely available Fortran application NODF [51] and Turnover [52]. Source code is available from WU by request.

For statistical inference of NODF, C-score, and beta_*P*_, we used a null model approach and compared the observed co-occurrence metric scores with those obtained from 1000 null matrices each that were randomized using a null model that resamples the matrix with placement probabilities proportional to observed total abundances of rows and columns (proportional abundance model [53]). This is a conservative null model that has been shown to account well for inherent site differences and unequal species colonization probabilities (the mass effect) that are not directly linked to the pattern of interest [24]. Note that this model is equivalent to a neutral model without dispersal limitation and speciation. To account for richness effects [54], raw FAD scores were compared to a null model in which the trait expressions for each single trait were randomly reshuffled among species. Statistical significance was estimated from the respective tail distributions at the two-sided 5% error level. Additionally, we calculated standardized effect sizes (SES = Obs—Exp) / StDev_Exp_; Obs and Exp: observed and expected scores, StDev_Exp_: standard deviation of expectation). SES scores should have values below –1.96 and above +1.96 at the two-sided 5% error level under the assumption that the respective null distribution is approximately normal. To account for multiple testing, all significance levels were Bonferroni corrected.

Possible competitive interactions among species within the seven feeding guilds were assessed following Ulrich et al. (2014) [31] and Soliveres et al. (2015) [32]. For each guild, we calculated 100,000 random species × species competitive strengths matrices, translated these into a column stochastic transition matrix, and used a Markov chain model to predict relative species abundances from this transition matrix within the 12 fragments. We compared predicted and observed relative species abundances by rank order correlation (r_C_) and chose the best fitting competition matrix to assess the maximum impact of interspecific competition on community assembly. High values of r_C_ point to the possibility that interspecific competition is a major driver for observed species distributions while low r_C_ values point towards a minor impact of competition [55].

**Fig 1:**
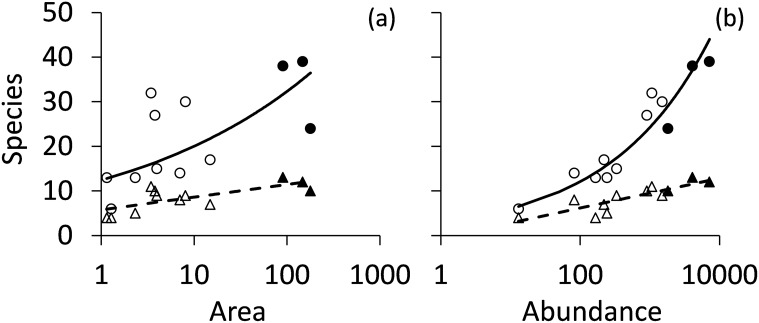
Species—area (a) and species—abundance (b) relationships of all species (circles) and of forest specialist species (triangles) of the East African Taita forest fragments. Power function ordinary least squares regressions to all species: a: S = 12.4A^0.21^, r^2^ = 0.44, P < 0.01; b: S = 3.03 I^0.30^, r^2^ = 0.92, P < 0.001. Logarithmic regression to specialist species a: S = 1.23 ln A + 5.78, r^2^ = 0.53, P < 0.01; b: S = 1.45 ln I– 0.49, r^2^ = 0.73, P < 0.01.

Using the empirical species—area relationship *S* = 12.4*A*^0.21^;*r*^2^ = 0.44 (Fig 1a) we assessed average species densities S_A_ per ha area by S_A_ = S/A^0.21^, with *S* reflecting the total species richness and *A* the fragment area. We used a general linear models with identity link function and normal error structure to relate FAD to the categorical variables feeding guild, functional traits, and remnant size group (large—small), and to species richness as the continuous co-variate.

## Results

We recorded a total of 17,520 individuals from 69 bird species in the 12 Taita forest fragments (Table 1), of which 36 species belonged to the insectivorous guild and 12 species to the frugi/nectarivorous guild (Table 2). Species richness increased moderately with area (Fig 1a) and perimeter (r = 0.61, P = 0.03). The best predictor of fragment richness was the total number of individuals caught (Fig 1b). Richness did not significantly vary with fragment isolation (Pearson r = 0.27, P > 0.3) and increased weakly with forest cover within the matrix (Pearson r = 0.51, P = 0.09). In fragments below 15 ha, species richness was independent of fragment area (Fig 1a, r = 0.17, P > 0.5) but tended to be positively related to fragment perimeter, albeit not statistically significant (r = 0.61, P = 0.08). Most species rich were the Ngangao (39 species) and Chawia (38 species) forest fragments, while the large and predominately pristine Mbololo fragment was comparatively poor in species richness (32 species). Of the smaller fragments, Fururu and Macha were most species rich (30 and 32 species, respectively).

**Table 1:** Area, perimeter, degree of isolation, percentage of closed-canopy forest cover within 800 m, and total number of species and individuals, of 12 indigenous forest fragments.

Fragment	Area (ha)	Perimeter (ha)	Isolation	Cover	All Species	Forest specialists	Individuals
**Mbololo**	178.79	9980	0.39	46.2	24	10	1797
**Ngangao**	146.93	11529	0.54	48.6	39	12	7179
**Chawia**	90.25	5291	0.37	42.0	38	13	4066
**Ronge**	14.81	2035	0.33	36.4	17	7	219
**Fururu**	8.04	1495	0.53	5.1	30	9	1492
**Vuria**	6.99	1099	0.18	18.0	14	8	78
**Yale**	3.94	897	0.56	6.1	15	9	330
**Ndiwenyi**	3.76	893	0.53	4.4	27	10	898
**Macha**	3.42	1728	0.56	2.1	32	10	1054
**Mwachora**	2.31	606	0.25	2.0	13	5	239
**Kichuchenyi**	1.28	514	0.53	1.1	6	4	13
**Wundanyi**	1.14	455	0.50	1.6	13	4	166

**Table 2:** Competition impact (r_C_), SES scores (proportional abundance null model) and species co-occurrences metrics (C-score, NODF, proportional species turnover beta) for three large and nine small East African forest fragments (cf. Table 1). Significant SES score (P < 0.05) are marked in bold. The single granivore forest specialist species made it impossible to calculate respective co-occurrence metrices.

Guild	Species (all)	Species (large)	Species (small)	Competition metric		SES scores					
**All species**											
				r_C_ (large)	r_C_ (small)	C-score (large)	C-score (small)	NODF (large)	NODF (small)	Beta (large)	Beta (small)
Frugi-/Nectarivores	12	8	10	0.10	0.40	**2.91**	-1.16	-1.41	0.15	**3.34**	1.63
Insectivores	36	33	26	0.56	0.19	1.42	1.72	**-3.01**	**-2.89**	**3.16**	**5.82**
Omnivores	11	10	10	0.71	0.36	-0.56	1.5	1.46	**-2.46**	0.48	**2.34**
Granivores	10	5	9	0.08	0.01	1.23	1.11	-0.74	-1.55	0.58	**4.24**
**Forest specialists**											
				r_C_ (large)	r_C_ (small)	C-score (large)	C-score (small)	NODF (large)	NODF (small)	Beta (large)	Beta (small)
Frugi-/Nectarivores	4	4	3	0.63	0.88	0.07	-0.45	-0.07	0.41	0.00	-0.22
Insectivores	10	9	7	0.78	0.55	0.05	0.98	0.19	0.67	-0.11	0.77
Omnivores	3	3	3	0.67	0.66	0.00	0.57	0.00	0.49	0.00	0.14

**Fig 2:**
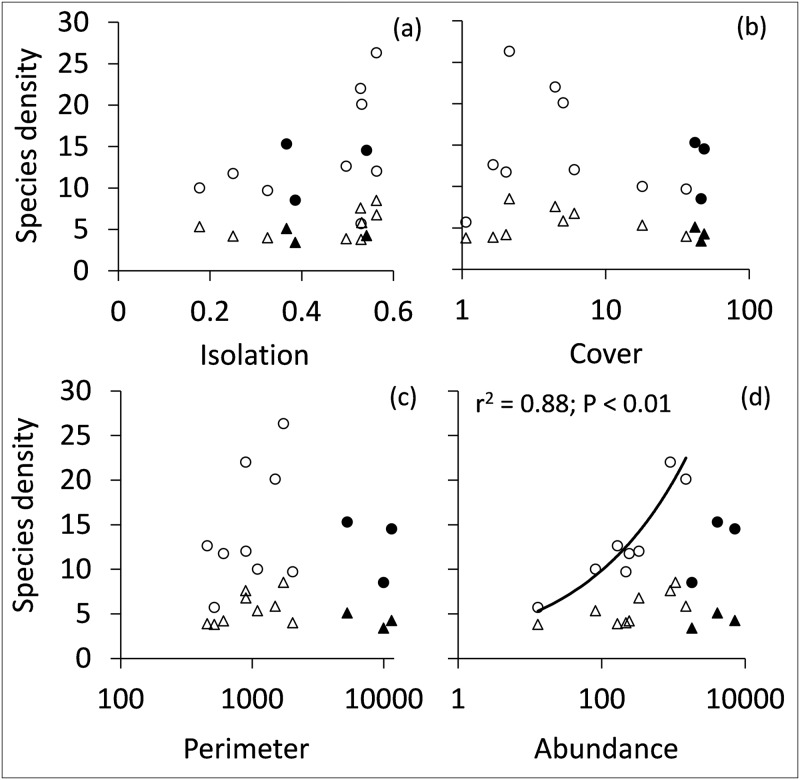
Bird species density per forest fragment of all species (circles) and of forest specialist species (triangles) was independent of fragment isolation (a), percentage of forest cover outside the fragments (b), and fragment perimeter (c), but increased with abundance in small fragments (d). coefficients of determination and associated parametric significance levels in (d) refer to a power function model.

Average species density was independent of fragment isolation (Fig 2a), forest cover (Fig 2b), and fragment perimeter (Fig 2c). In the smaller fragments, species density increased with total abundance (Fig 2d). In the larger fragments, species density was at an intermediate level compared to the smaller fragments (Fig 2d). A comparison of the relative abundances between the smaller and the larger fragments revealed a significant shift in relative abundance between the two fragment types. Of the 22 species with relative abundances below 0.001 in the larger fragments, 18 achieved higher relative abundances in the smaller fragments (not shown). This shift was accompanied by a sharp decline of five species (*Columba larvata*, *Phyllastrephus placidus*, *Phylloscopus ruficapillus*, *Turdus olivaceus*, *Zoothera gurneyi*) in the smaller fragments.

A separate analysis based on forest specialist species only (Table 1) also revealed an increase in richness with fragment size (Fig 1a) and abundance (Fig 1b), however, less strong so compared to an analysis with all species included (Fig 1). The proportion of forest specialists decreased with fragment area (r = -0.32, P = 0.32) and abundance (r = -0.73, P < 0.01), while specialist species density was not significantly related to habitat isolation (Fig 2a), forest cover (Fig 2b), fragment perimeter (Fig 2c), or specialist abundances (Fig 2d).

A comparison of species richness between the three larger (total area 416 ha, 56 species) and nine smaller fragments (total area 46 ha, 55 species) (Table 1) revealed a loss of 14 species (25%) and a gain of 13 species (23.6%) in the latter. The larger fragments contained 17 species of forest specialists, the smaller ones 14 species. Despite the fact that the nine smaller fragments comprised only 11% of the area of the larger fragments, total species richness decreased by 2% (1 species) only. The prevalence of negative nestedness and positive beta_*P*_ SES scores (Table 2) further indicated spatial turnover in species composition among the larger and smaller fragments, however, we did not find direct evidence that the latter was caused by competitive interactions. Indeed, correlations between the competitive interaction matrix and the observed distribution of abundances (Table 2) were on average weak and explained at most 50% of variance in abundance.

**Fig 3:**
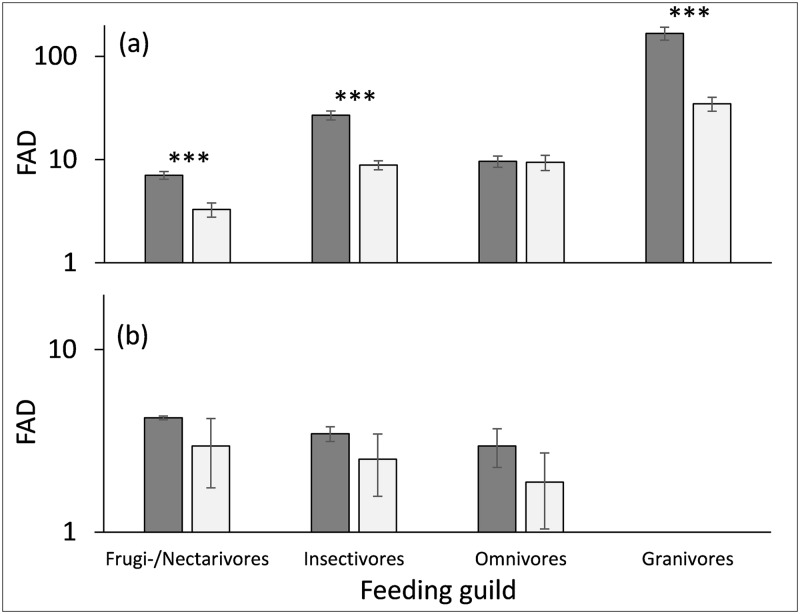
Functional attribute diversity (a: all species, b: forest specialists only) of four bird feeding guilds differed significantly (***: parametric P(F) < 0.001) between large (dark grey columns) and small fragments (light grey columns dots). The single granivore forest specialist species made it impossible to calculate FAD.

Finally, we compared functional diversity between feeding guilds and between larger and smaller fragments (Fig 3a, Table 3). Except for omnivores, functional attribute diversity significantly differed between feeding guilds and was always larger in the large fragments (Fig 3a). We observed the same pattern for forest specialists (Fig 3b) although the differences were statistically not significant due to the small number of species. Linear modelling accounting for differences in species richness between fragments (Table 3) revealed that these differences were mainly caused by co-variation with species richness. Although FAD significantly differed between feeding guilds and guild specific differences in species traits (Table 2), FAD did not vary with fragment size (Table 3). FAD also did not significantly vary with any of the categorical variables (guild, trait, size) when using standardized effect scores (Table 3).

**Table 3:** General linear mixed modelling detected significant (*: parametric P(F) < 0.05; ***: P < 0.001) differences of functional attribute diversity (FAD) among feeding guilds, and guild×functional trait combinations when using raw FAD as dependent variable, but not when using SES transformed values (traits reshuffling null model). Fragment species richness served as metric covariate. Given are partial eta^2^ values.

Variable	df	FAD	SES FAD
**Feeding guild**	3	0.06***	0.03
**Functional trait**	5	0.02	0.02
**Remnant size**	1	<0.01	<0.01
**Guild×trait**	15	0.11***	0.09
**Guild×size**	3	0.04	<0.01
**Trait×size**	5	0.03	0.03
**Species**	1	0.04*	<0.01
**Squared species**	1	0.65***	<0.01
**Error**	229		
**r**^**2**^ **(model)**		0.95***	0.18

## Discussion

Within an island biogeographic framework [56], species richness is predicted to vary positively with patch size and negatively with patch isolation. Along these lines, Canale et al. (2012) [57] reported a strong decline in species richness in tropical forest fragments of less than 10 ha (equivalent to a lowered species density), approximately the upper size limit of our small forest fragments. Hanski et al. (2013) [58] predicted such a decline while demonstrating that SARs that only account for area effects tend to overestimate species richness in fragmented landscapes if fragments are highly isolated, and they proposed an extension of the power function SAR that downsizes species richness in small fragments. In turn, a number of empirical studies reported a small island effect [12] where species density in very small islands or habitat patches increased [59–60]. Our results do not support either prediction. Species richness in the smallest fragments did not negatively deviate from the observed SAR (Fig 1a) although we observed a tendency to independence of area below 10 ha. However, richness was very closely related to the number of individuals indicating that habitat capacity is probably the main trigger of species richness. Contrary to Hanski et al. (2013) [58], area corrected richness (species density) was not linked to fragment isolation (Fig 2a).

The counter-intuitive finding that species richness in small fragments was only 2% lower than in large ones, is in line with other studies that reported patch connectivity to be of higher importance than patch size [61], in particular for generalist species with a broad ecological amplitude that can easily cross the landscape matrix and (re)colonise small forest patches [62, 63]. This generalist-focused explanation is corroborated by the fact that specialist species increased less in richness in the larger fragments (Fig 1). Bird mobility in heterogeneous landscapes tends to vary among feeding guilds [64–66], with small understorey insectivores often showing the highest sensitivity to fragment isolation [67]. The resulting high species turnover (i.e. partial replacement of sedentary, forest-restricted specialists by mobile, matrix-tolerant generalists in small, degraded forest fragments) might be responsible for the weak SAR observed in our study.

Previous studies showed that bird assemblages strongly vary in composition with habitat area and structure (such as the degree of fragmentation), and predicted higher species richness in large, connected forest patches [68–69]. Yet, Trzinski et al. (1999) [70], Banks-Leite et al. (2012) [62], and Neuschulz et al. (2013) [71] documented no significant impact of habitat heterogeneity on bird community structure in tropical forest fragments. Banks-Leite et al. (2012) [62] argued that loss of tropical species is mainly driven by habitat destruction, rather than fragmentation. According to these authors, species with broad ecological amplitude can readily exploit the landscape matrix in which forest fragments are embedded and may even gain from habitat fragmentation. The high species turnover between isolated forest fragments (independent of fragment size) observed in our study is in line with these predictions.

Various studies have reported increasing mobility with decreasing habitat integrity [72–74]. Yet, Price (2006) [75] showed that frugivorous species conducted shorter, rather than longer, movements in fragmented forests, despite their high intrinsic mobility. Based on earlier studies of mobility [76–77] and gene flow [78] in the same study area, bird species currently surviving in the Taita Hills forest also appear to differ in metapopulation dynamics. Results of this study add to the effect of functional connectivity [79] and support the need to define species- (or guild-) specific fragmentation thresholds [80] in conservation. Indeed, even though we found species richness to be only marginally affected by patch area, there are documented cases of local species extinctions in small Taita forest fragments [42]. In this respect, our results are also relevant for the ongoing SLOSS debate [81–82]. Although each large fragment was at least twice as large as all smaller fragments together, they hosted at most 75% of the total species richness of the small fragments (Table 2). As such, our findings corroborate the theoretical expectation of Lasky & Keitt (2013) [26] that networks of small habitat remnants would be able to maintain higher total species richness than homogenous habitat blocks of the same size. Yet, contrary to these authors, we did not find reduced fragment (alpha) diversities within each of the smaller fragments. We interpret these findings as evidence that tropical forest birds respond to increased habitat fragmentation by higher mobility [78], or that there is a debt between former habitat destruction and ongoing local extinction [83].

We were surprised to see that functional trait identity was not linked to the pattern of species—co-occurrence. Species interactions are mediated by traits and classical community assembly theory predicts co-existing species to show low levels of trait similarity [84]. Yet, we did not find strong evidence that the bird communities in our study fragments are shaped by competitive interactions (Table 2), which might explain why trait expression was not significantly linked to co-occurrence. This finding contrasts to recent evidence by Bregman et al. (2015) [16] who reported increased levels of interspecific competition within bird guilds at decreasing fragment size. However, these authors used indirect evidence within the community assembly framework that assumes that overdispersion of phylogenetic and functional traits is a consequence of competitive interactions (Darwin’s competition-relatedness hypothesis, reviewed in Allan et al. 2013, Götzenberger et al. 2012) [85–86]. However, Cahill et al. (2008) [87] found little evidence for this assertion and it is now well known that overdispersion (spatial segregation) might stem from different processes, particularly from filter effects within heterogeneous landscapes [88] and even from dispersal limited neutral community assembly [89]. Here we used a direct way to assess whether any set of competitive strength relationships between species is able to predict observed abundance distributions. For the smaller fragments this relation was highest in omnivores where competition explained at most 13% of variance in species relative abundances (r_C_ = 0.36, Table 2) comparing to 50% in the larger fragments. Apparently species density in the smaller fragments yet did not reach the threshold for intense competitive effects and thus is of rather marginal importance for community assembly.

Total trait space, as expressed by FAD, increases with species richness [52]. However, various authors found homogenization effects in fragmented landscapes where smaller fragments are devoid of habitat specialists and regionally rare species [90–92]. Consequently, this selective pattern of species extinction should translate into a reduced effective functional diversity that is the degree of FAD after accounting for richness effects. Rather than observing such a pattern, FAD remained constant after correction for richness differences (Table 3). While small tropical forest fragments are hence able to maintain a high effective functional diversity, communities will inevitably collapse when fragment areas become exceedingly small, which raises the question about a minimal tropical forest fragment size. SES-transformed FAD scores of the three smallest fragments (Mwachora, Kichuchenyi, Wundanyi, Table 1) were indeed smaller (average SES FAD = -0.26±0.14) than those of the three large fragments (average SES FAD = 0.13±0.15), although not statistically significant. A one ha area is probably at the lower boundary for a functioning understorey bird community in the Taita forest archipelago.

### Conclusion

In conclusion, results of this study do not point to a single ecological driver of the observed variation in avian species composition among indigenous fragments of the Taita forest archipelago. Interspecific competition does not appear to be a major driver of species segregation, since in small forest fragments no single competitive strength hierarchy was able to predict at least a major part of the observed species abundances distributions. In larger fragments, however, the relative importance of competition might be higher. We did not find strong evidence for habitat filtering either, and community-wide species co-occurrences and joint occurrences between pairs of species were neither nested nor segregated, as would be expected if competition would be the main driver of species occurrences. The frequency of pairwise species segregation was even much below the level expected under random association. However, the large differences in species density and species richness between the two smallest fragments highlight that local peculiarities might heavily constrain species richness, irrespective of subsequent patterns of co-occurrences. Such variable, and partly opposing, responses of single guilds to fragmentation suggest that it is vital to take species ecology into consideration when predicting community-wide responses to habitat change in tropical forests.

## Supporting Information

S1 Appendix Location of our study region, the Taita Hills in southern Kenya (map A), and the detailed map of the forest fragments from where bird observations were available (map B), including the following 12 forest patches: Mbololo (1), Ronge (2), Ngangao (3), Vuria (4), Yale (5), Wundanyi (6), Mwachora (7), Macha (8), Kichuchenyi (9), Ndiwenyi (10), Fururu (11), Chawia (12). (XLSX)
